# A Phase 2 Trial of Ibrutinib and Nivolumab in Patients with Relapsed or Refractory Classical Hodgkin’s Lymphoma

**DOI:** 10.3390/cancers15051437

**Published:** 2023-02-24

**Authors:** Walter Hanel, Polina Shindiapina, David A. Bond, Yazeed Sawalha, Narendranath Epperla, Timothy Voorhees, Rina Li Welkie, Ying Huang, Gregory K. Behbehani, Xiaoli Zhang, Eric McLaughlin, Wing K. Chan, Jonathan E. Brammer, Samantha Jaglowski, John C. Reneau, Beth A. Christian, Basem M. William, Jonathon B. Cohen, Robert A. Baiocchi, Kami Maddocks, Kristie A. Blum, Lapo Alinari

**Affiliations:** 1 Division of Hematology, Department of Medicine, The Ohio State University, 460 W 10th Ave., Columbus, OH 43210, USA; 2 Center for Biostatistics, Department of Biomedical Informatics, The Ohio State University, Columbus, OH 43210, USA; 3 Blood and Marrow Transplant and Cell Therapy Program, OhioHealth, 500 Thomas Ln #A3, Columbus, OH 43214, USA; 4 Department of Hematology and Medical Oncology, Winship Cancer Institute, Emory University, 1365 Clifton Road NE, B4013, Atlanta, GA 30322, USA

**Keywords:** Hodgkin’s lymphoma, nivolumab, ibrutinib

## Abstract

**Simple Summary:**

Relapsed or refractory classical Hodgkin lymphoma remains a difficult treatment challenge. Despite responses with the checkpoint inhibitors nivolumab and pembrolizumab, patients eventually progress. Combining other treatments with checkpoint inhibitors may provide more frequent and durable responses in this setting. We conducted a phase II study in relapsed or refractory Hodgkin lymphoma combining nivolumab with the Bruton’s tyrosine kinase inhibitor ibrutinib. Although we did not find an increase in the response rate of this combination compared to that previously reported, responses tended to be durable even in patients who progressed on nivolumab therapy prior to enrollment. Larger studies combining Bruton’s tyrosine kinase inhibitors with checkpoint blockade are warranted, especially in patients who had progressed previously on checkpoint inhibitor therapy.

**Abstract:**

Background: Relapsed or refractory classical Hodgkin lymphoma (cHL) remains a difficult treatment challenge. Although checkpoint inhibitors (CPI) have provided clinical benefit for these patients, responses are generally not durable, and progression eventually occurs. Discovering combination therapies which maximize the immune response of CPI therapy may overcome this limitation. We hypothesized that adding ibrutinib to nivolumab will lead to deeper and more durable responses in cHL by promoting a more favorable immune microenvironment leading to enhanced T-cell-mediated anti-lymphoma responses. Methods: We conducted a single arm, phase II clinical trial testing the efficacy of nivolumab in combination with ibrutinib in patients ≥18 years of age with histologically confirmed cHL who had received at least one prior line of therapy. Prior treatment with CPIs was allowed. Ibrutinib was administered at 560 mg daily until progression in combination with nivolumab 3 mg/kg IV every 3 weeks for up to 16 cycles. The primary objective was complete response rate (CRR) assessed per Lugano criteria. Secondary objectives included overall response rate (ORR), safety, progression free survival (PFS), and duration of response (DoR). Results: A total of 17 patients from two academic centers were enrolled. The median age of all patients was 40 (range 20–84). The median number of prior lines of treatment was five (range 1–8), including 10 patients (58.8%) who had progressed on prior nivolumab therapy. Most treatment related events were mild (<Grade 3) and expected from the individual side effect profiles of ibrutinib and nivolumab. In the intent to treat population (*n* = 17), the ORR and CRR were 51.9% (9/17) and 29.4% (5/17), which did not meet the prespecified efficacy endpoint of a CRR of 50%. In patients who received prior nivolumab therapy (*n* = 10), the ORR and CRR were 50.0% (5/10) and 20.0% (2/10), respectively. At a median follow up of 8.9 months, the median PFS was 17.3 months, and the median DOR was 20.2 months. There was no statistically significant difference in median PFS between patients who received previous nivolumab therapy versus patients who were nivolumab naïve (13.2 months vs. 22.0 months, *p* = 0.164). Conclusions: Combined nivolumab and ibrutinib led to a CRR of 29.4% in R/R cHL. Although this study did not meet its primary efficacy endpoint of a CRR of 50%, likely due to enrollment of heavily pretreated patients including over half of who had progressed on prior nivolumab treatment, responses that were achieved with combination ibrutinib and nivolumab therapy tended to be durable even in the case of prior progression on nivolumab therapy. Larger studies investigating the efficacy of dual BTK inhibitor/immune checkpoint blockade, particularly in patients who had previously progressed on checkpoint blockade therapy, are warranted.

## 1. Introduction

Although classical Hodgkin’s lymphoma (cHL) is a generally curable disease with combination chemotherapy, treatment of patients who relapse after chemotherapy remains a difficult challenge [1,2]. Autologous stem cell transplant (auto-HCT) continues to be the standard of care for patients with chemo-sensitive relapse and who are able to tolerate further treatment [3]. For patients who relapse after auto-HCT or who are unable to tolerate auto-HCT, targeted treatment options including anti-CD30 therapy with brentuximab or checkpoint inhibitors (CPI) with either nivolumab or pembrolizumab have improved patient outcomes [4,5,6,7,8].

Nivolumab is a monoclonal antibody that inhibits programmed death receptor 1 (PD1), a negative regulatory receptor of T-cells, leading to more effective immune-mediated antitumor responses in many tumor types including cHL [5,6,7]. Patients treated with nivolumab after relapsing after an auto-HCT on the Checkmate-205 study had an overall response rate (ORR) and complete response rate (CRR) of 69% and 16%, respectively, with a median progression free survival (PFS) of 14.7 months while treatment with pembrolizumab on the KEYNOTE-087 study showed an ORR and CRR of 71.9% and CRR of 27.6% with a PFS of 13.7 months [5,6,7]. More recently, data from the KEYNOTE-204 trial randomizing patients either postauto HCT or who were ineligible for auto-HCT to either pembrolizumab or brentuximab showed superiority for pembrolizumab with an ORR, CRR, and median PFS of 65.6%, 25%, and 13.2 months [8]. Despite the progress that has been made with CPI monotherapy in the relapsed setting, patients will eventually progress on therapy with no effective available treatment options after failure of both CPI and anti-CD30 therapy aside from allogeneic-HCT (allo-HCT), which is feasible in only selected patients. Thus, new approaches to maximize responses to CPI therapy may significantly increase the depth and durability of responses and further improve patient outcomes.

Bruton tyrosine kinase inhibitor (BTKi) therapy has revolutionized the treatment of chronic lymphocytic leukemia (CLL) and has also been found to be effective in certain types of B-cell non-Hodgkin’s Lymphoma (NHL) [9]. Aside from direct cytotoxic effects, BTKi therapy has been shown to have immune modulatory properties [10]. Patients with CLL treated with ibrutinib after 8 weeks have increased CD8 cells with increased effector T cells to Treg ratios [11]. Longer-term treatment of ibrutinib may reverse T-cell exhaustion by reducing PD-1 expression on chronically activated CD8 T-cells and reconstitution of T cell cytokine production [12,13]. In addition, ibrutinib is known to potentiate Th1-mediated immune responses through inhibition of interleukin-2–inducible kinase (ITK) [14]. However, ibrutinib can also suppress NK-cell-mediated cytotoxicity and suppresses TLR-induced phagocytosis of tumor cells by monocytes [15,16,17], indicating potentially mixed effects on different immune subtypes. Immunohistochemistry for BTK has shown staining in the immune cells within the cHL microenvironment without staining the Reed Sternberg (RS) cells themselves [18], suggesting that BTKi therapy in cHL may play a role in modulation of the immune microenvironment rather than being directly cytotoxic to RS cells themselves. On the other hand, the src family kinases Lyn, Fyn, and Syk, which are expressed in RS cells and are known to be inhibited as an off-target effect by ibrutinib, lead to potential direct RS cytotoxicity in addition to the immunomodulatory effects discussed above [19].

Initial case reports of two patients with heavily pretreated cHL in the post allo-HCT period treated with ibrutinib showed a near CR in one patient who eventually progressed at 4 months and an ongoing CR in another patient out to 6 months [20]. In a case series of seven heavily pretreated cHL patients including three patients who relapsed after allo-HCT treated with single-agent ibrutinib, four patients responded, three of which had CRs and two of which were still ongoing at 3 and 15 months [21]. A phase II trial (NCT02824029) evaluating ibrutinib monotherapy in R/R cHL is currently ongoing.

Based on the possible T-cell and tumor microenvironment effects of ibrutinib as well as the monotherapy activity of patients with relapsed cHL, we investigated whether combination therapy with ibrutinib and nivolumab would lead to deeper and more durable responses in R/R Hodgkin’s lymphoma.

## 2. Materials and Methods

### 2.1. Study Design and Patients

This was a single arm phase II trial of nivolumab plus ibrutinib (NCT02940301) conducted across two centers (The James Comprehensive Cancer Center and Emory University Hospital). Patients 18 years of age or older with cHL who received at least one prior treatment were enrolled. Patients were allowed to have received prior CPI, but patients could not have received prior ibrutinib. Prior auto-HCT was not required but permitted, while prior allogeneic-HCT was excluded. Patients were allowed to come off early to undergo either auto-HCT or allo-HCT at the discretion of the treating physician.

### 2.2. Treatments

Nivolumab (3 mg/kg) was given on day 1 of a 21-day cycle while Ibrutinib (560 mg) was given continuously on days 1–21. Nivolumab was continued until disease progression for a maximum of 16 cycles. Ibrutinib was continued until disease progression. All patients were started at dose level (DL) 0 (nivolumab 3 mg/kg, ibrutinib 560 mg), and dose reductions for toxicity were performed according to the following dose levels: DL-1, nivolumab 2 mg/kg, ibrutinib 420 mg; DL-2, nivolumab 1 mg/kg, ibrutinib 280 mg).

### 2.3. End Points and Assessments

The primary endpoint of this study was complete response rate (CRR) as assessed with CT or CT-PET per Lugano criteria [22]. Imaging was performed prior to cycles 4, 7, 10, and 16 and every 8 cycles thereafter. Secondary endpoints include overall response rate (ORR), progression free survival (PFS), duration of response (DOR), and toxicity. Patients who withdrew early due to toxicity or disease progression prior to disease response assessment were included in the denominator when calculating the CRR and ORR as part of the intent to treat population. Patients coming off trial early to undergo an HCT were censored at the time of transplant. The data cutoff for this study was 5/1/2022.

### 2.4. Immune Phenotyping by CyTOF

Blood was collected from the study subjects on Cycle 1 Day 1 prior to administration of the first dose of treatment, and on Cycle 4 Day 1. Whole blood was fixed using proteomic stabilization buffer (Smart Tube PROT-1). Fixed blood was stored at −80 °C. Prior to staining, fixed blood was defrosted. Red blood cells were lysed in water. PBMCs were recovered and washed in phosphate buffer saline, followed by cell staining buffer (CSB; Fluidigm 201068). Two million fixed PBMCs were used per staining reaction. Surface and intracellular antigen staining was performed using standard techniques. Briefly, Fc-blocked cells were resuspended in 50 μL of cell staining buffer. An amount of 50 µL of the surface marker antibody cocktail was added to each tube (final volume 100 µL). Samples were shaken for 50 min at room temperature. After surface staining, cells were washed with CSB, fixed in 1.5% paraformaldehyde, and permeabilized with −20 °C methanol for 15 min. Subsequently, cells were washed in PBS and CSB and stained with intracellular antibodies at room temperature with shaking, in a final volume of 100 µL. Following additional wash steps, cells were fixed in PBS containing 1.5% paraformaldehyde and a 1:5000 dilution of the iridium intercalator pentamethylcyclopentadienyl-Ir(III)-dipyridophenazine (Fluidigm, San Francisco, CA, USA) for 12–26 h. Excess intercalator was washed away prior to data collection. Data was collected using a Helios^TM^ mass cytometer (Fluidigm) at a rate of 200–400 events per second. Events in the amounts of 600,000–1,000,000 per sample were collected. Data visualization and data analysis were performed on the cloud-based platform Cytobank (Cytobank, Inc., Brea, CA, USA). Cell populations of interest were identified using manual gating. For a more detailed description of cyTOF methods, see the supplemental section.

Antibody clones and vendors, as well as metal conjugates, are listed in Table S7. Metal-labeled antibodies were purchased from Fluidigm. Antibodies purchased from other vendors were conjugated to metals purchased from Fluidigm according to the manufacturer’s instructions using the multimetal labeling kit (Fluidigm 201300).

### 2.5. Statistics

To determine target enrollment, Simon’s two-stage design was used to test the null hypothesis that the true CR rate is <20% versus the alternative hypothesis that the true CR rate is >50%. With Type I and II errors constrained to 0.10, ten patients were initially enrolled. Of these patients, 3 patients achieved a CR (30%), warranting expansion of the total enrollment to 17 patients. PFS and DOR data were analyzed by Kaplan–Meier method with significance determined by log rank analysis.

For cyTOF analyses, comparisons were performed using unpaired t-test to detect significant differences in immune cell population prevalence between responders and nonresponders, followed by false discovery rate adjustment. Data analysis was performed using a combination of manual gating using Cytobank software. Principal component analysis was performed to assess clustering of responding patients compared to nonresponding patients. The first and second components were plotted for the various immune expression populations at Cycle 1, and T cell profiles at Cycle 1, Cycle 4, and Cycles 12–16. Individual expression levels were compared by response using Wilcoxon rank-sum tests. Results of two-sample t-tests are also presented given the low power of Wilcoxon tests with small sample sizes. Due to the exploratory nature of these analyses, *p*-values were not adjusted for multiple comparisons. Statistical analyses were performed using SAS version 9.4 (SAS Institute Inc., Cary, NC, USA).

## 3. Results

### 3.1. Patient Baseline Characteristics

A total of 17 patients were enrolled between the dates of 3/2017 and 3/2021 (Table 1). The median age was 40, with 5 patients >60 years of age. Patients received a median of 5 prior therapies (range 1–8), including brentuximab in 76.5% and nivolumab in 58.8%. Eight of ten patients previously treated with nivolumab progressed while receiving nivolumab. Of the remaining 2 patients, one patient had stable disease while on therapy, and the other patient completed 12 cycles of maintenance post auto-HCT. The median time from the last CPI was 4.9 months. Eight patients (47.1%) underwent a prior auto-HCT.

### 3.2. Efficacy

**Figure 1:**
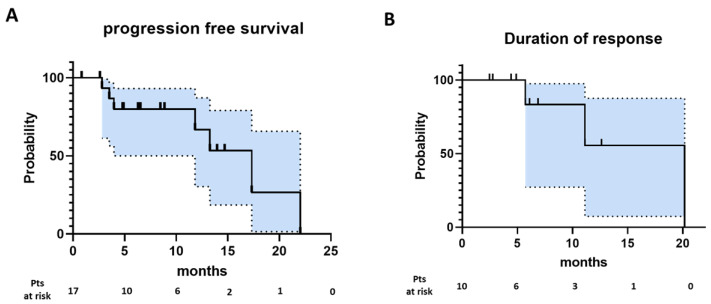
Kaplan–Meier curve for progression-free survival (**A**, *n* = 17) and duration of response (**B**, *n* = 10) are shown.

The ORR and CRR of all patients in the intent to treat population (*n* = 17) was 52.9% (95% CI, 31.0–73.8%) and 29.4% (95% CI, 12.9–53.4%), respectively (Table 2). Seven patients achieved their best response on first disease assessment (prior to Cycle 4) while two achieved a deeper response with further therapy: One patient converted from a PR to a CR at the second response assessment while the other patient converted from SD to a PR. With a median follow up of 8.9 months, the median PFS was 17.3 months with a median DOR of 20.2 months (Figure 1).

**Figure 2:**
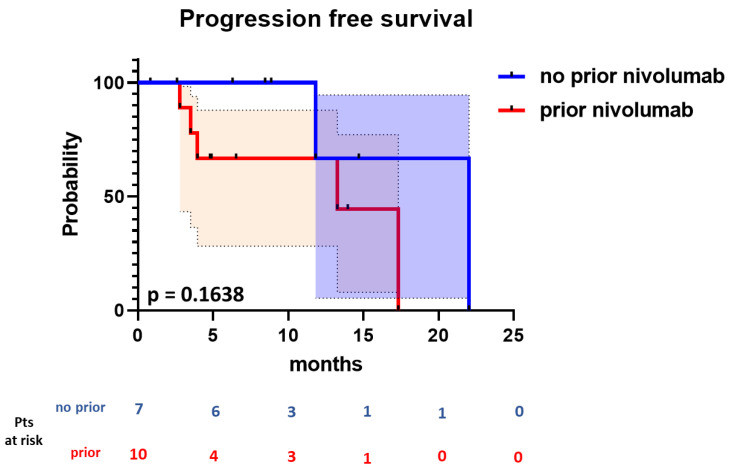
Progression-free survival stratified into nivolumab naïve patients (blue line) and patients with prior nivolumab (red line).

When stratifying patients according to previous nivolumab, the ORR and CRR for nivolumab naïve patients (*n* = 7) was 57.1% (95% CI, 25.0–84.2%) and 42.8% (95% CI, 15.7–75.0%). In patients treated with prior nivolumab (*n* = 10), the ORR and CRR were 50% (95% CI, 23.7–76.3%) and 20% (95% CI, 0–44.8%). The majority of patients receiving prior CPI therapy had progressed while on therapy (*n* = 8 of 10). Five of these patients with prior progression responded to the combination of nivolumab and ibrutinib, and two of these five achieved a CR. There was no significant difference in the median PFS (22.0 months vs. 13.3 months) between patients with prior nivolumab and patients who were nivolumab naïve (*p* = 0.164) (Figure 2).

**Figure 3:**
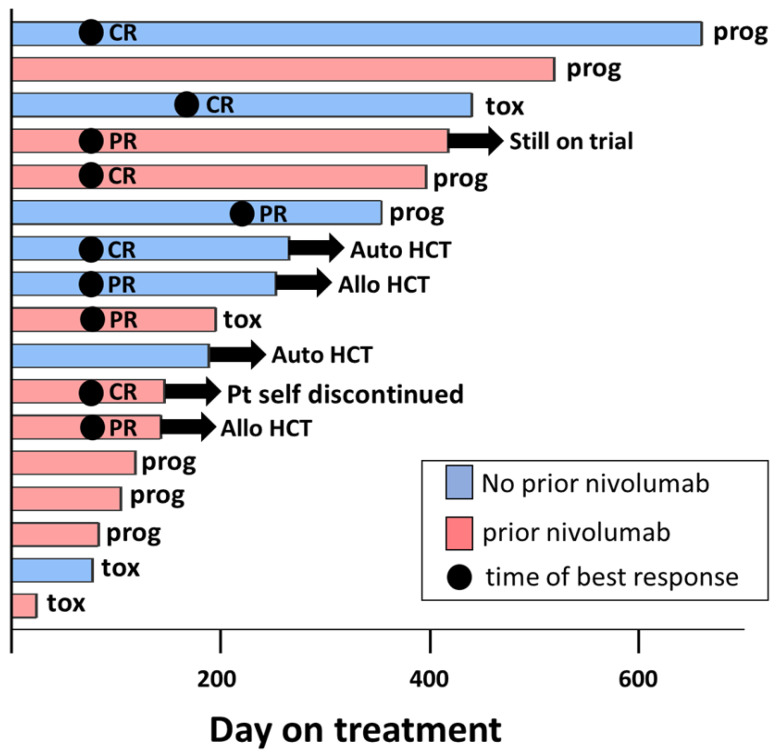
Swimmer’s plot of all patients enrolled in trial.

Four patients came off trial to receive HCTs (*n* = 2 auto, *n* = 2 allo). Of the patients that underwent auto-HCT, the first patient had stable disease at the time of coming off trial. This patient underwent ICE chemotherapy followed by auto-HCT and converted into CR post-HCT. This patient remained in CR at the time of data cutoff. The second patient achieved a CR on trial and proceeded directly to auto-HCT without chemotherapy and remained in CR at the time of data cutoff. Of the patients that underwent allo-HCT, the first patient had a CR at the time of transplant and had no evidence of acute graft versus host disease (GVHD) in the post-transplant period but did eventually relapse one year after transplant and went on an alternate therapy. Their last nivolumab infusion was 57 days prior to their allo-HCT. The second patient had a partial response at the time of HCT and developed severe acute GVHD involving the skin, liver, and colon in the post-HCT period, which persisted as chronic GVHD. This patient received their allo-HCT 42 days after receiving their last nivolumab infusion. The patient was disease-free post allo-HCT but eventually died due to complications of chronic GVHD. A swimmer’s plot summarizing the outcome of each individual patient enrolled is shown in Figure 3.

### 3.3. Safety

Most treatment-related events were mild (<Grade 3) and expected from the individual side effect profiles of ibrutinib and nivolumab (Table 3). Four patients did have to discontinue treatment due to treatment-related side effects. One of these patients had persistent grade 2 LFT elevation despite holding therapy, another patient had a grade 3 rash, and the third patient had grade 3 hematuria. Biopsies were not acquired to further clarify if the etiology of these side effects was in fact immune related. Due to the overlapping side effect profiles of the individual drugs, specific relation to either ibrutinib or nivolumab could not be definitively concluded in these cases. The last patient came off trial after having sepsis with an associated pericardial effusion. In each of these cases, the side effect did resolve with treatment discontinuation. None of the patients enrolled on trial required treatment with high-dose steroids for side effect resolution.

### 3.4. Exploratory Immune Phenotyping

Immune phenotyping of T cell populations in fixed whole blood from available samples (nine responders and five nonresponders) was performed with mass cytometry. Twenty CD4+ and 20 CD8+ T cell subsets, including cells expressing cytotoxic molecules (granzyme B and perforin), transcription factors (T-bet, GATA3, and FoxP3), naïve and memory markers (CD45RA, CD45RO, and CD62L), markers of activation (HLA-DR and CD28), degranulation (NKG2D), immunomodulatory/apoptosis inducer (CD95, also known as Fas), exhaustion (LAG3 and Tim-3) and anergy (CD57), and checkpoint molecules (PD-1, CTLA4, PD-L1, and PD-L2) were evaluated.

Due to the interference of nivolumab treatment with effective binding of the PD-1 targeting antibody clone EH12.2H7 that was used in this study, which has been described elsewhere [23], we were not able to investigate the true proportion of circulating PD-1+ T cells in subjects after initiation of nivolumab treatment. Furthermore, many of the subjects were exposed to nivolumab before enrollment on this trial, which, in some cases, affected our ability to detect PD-1 expression. We selected a group of subjects that had not received nivolumab within 200 days of beginning the trial for analysis of PD-1 expression on Cycle 1, Day 1 (Table S1), and PD-1 was detected on the surface of multiple circulating T cell subsets (Table S1). We observed no significant differences in the expression of PD-1 in various T cell populations between responders and nonresponders in this subset of patients (Tables S1–S3, Figure S1). Further investigation of subpopulations of CD4+/PD-1+ and CD8+/PD-1+ T cells also showed no significant differences (Tables S2 and S3, Figure S1). Exploratory analysis across all markers revealed that nonresponders had a higher median percentage of CTLA4 (exhaustion marker) expression in CD4+/PD-1+ T cells although these results were not statistically significant after adjustment for multiple comparisons (Table S4, Figure S2).

Exploratory analysis of the prevalence of various circulating T cell subsets between responders and nonresponders before and after initiation of treatment that included all subjects revealed no differences on Cycle 1, Day 1 and Cycle 4, Day 1, between responders and nonresponders, after adjusting for multiple comparisons (Tables S4–S6). Given the known Th1 expansion induced by ibrutinib, we performed a focused analysis of the baseline and the Cycle 4, Day 1 populations of Th1 (CD4+Tbet+) and Th2 (CD4+GATA3+) subsets between responders and nonresponders (Figure S3). We found a higher baseline percentage of Th1 cells in responders that did slightly increase at Cycle 4, while nonresponders had lower levels of baseline Th1 cells that slightly decreased with treatment (Figure S3, left). Conversely, Th2 cells decreased in responders while increasing in nonresponders (Figure S3, right). However, both the average baseline levels of both T-cell subsets as well as the changes from Cycle 1 to Cycle 4 were not statistically significant between responders and nonresponders.

## 4. Discussion

In these heavily pretreated patients with a median number of prior lines of treatment of five, including 76.5% who received prior brentuximab, they achieved an ORR and CRR of 52.9% and 29.4%, respectively, which did not meet the prespecified efficacy endpoint of a CRR of 50%. The median PFS of all patients was 17.3 months. Therapy was generally well tolerated and toxicities that were encountered were anticipated based on the individual side effect profiles of ibrutinib and nivolumab. There was no appreciable increase in immune-related adverse events compared to what would be anticipated with nivolumab monotherapy, with no patients requiring treatment with high-dose steroids.

There is significant interest in improving upon the response rate and duration of response of CPI therapy in patients with relapsed cHL who either fail auto-HCT or are unable to tolerate auto-HCT. Several trials have been conducted to date exploring combination therapies with nivolumab to achieve improved responses both in the second line setting and in the multiply relapsed setting, including brentuximab, ICE chemotherapy, ipiliumumab, and brentuximab/ipililumamb [24,25,26,27]. In this phase II study, we investigated if ibrutinib, with its known immunomodulatory activity, could improve upon the responses of nivolumab therapy in patients with relapsed cHL. This combination has also been investigated in other B-cell malignancies including relapsed/refractory CLL with or without Richter’s transformation and other B-cell NHLs with activity similar to single-agent ibrutinib in the B-cell NHL cohort. However, the combination of ibrutinib and nivolumab resulted in a promising ORR of 65% with two CRs and 11 PRs in the Richter’s transformation cohort [28]. All patients in this study were ibrutinib- and CPI-naïve. A more recent phase II study of patients with Richter’s transformation enrolled patients with and without prior exposure to a Btk inhibitor with lower responses (64% vs. 23%) between Btk-naïve vs. Btk-exposed patients [29]. Our study was distinct in allowing patients with prior CPI therapy to be enrolled, which allowed us to also study whether the addition of ibrutinib could lead to durable responses in patients who have previously progressed on CPI.

When stratifying patients further by prior CPI use, we found an ORR and CRR for CPI-naïve patients (*n* = 7) of 57.1% and 42.8%, respectively, and 50% and 20% for patients who received previous CPI (*n* = 10). There is published retrospective data demonstrating the efficacy of CPI retreatment in relapsed/refractory cHL patients [30,31]. In the first study, seven patients who initially received either a CR or PR to nivolumab went on to achieve an ORR and CRR of 100% and 57.1%, respectively [27]. In the second study, a series of 23 patients who achieved a CRR to prior nivolumab therapy went on to achieve an ORR and CRR of 67% and 33.3%, respectively [28]. However, as patients in these studies had discontinued anti-PD1 therapy because of durable responses while on therapy, data on responses in the setting of CPI reintroduction in patients who had previously progressed on therapy is lacking. Our results do suggest that combination therapy with nivolumab and ibrutinib may possibly resensitize patients to checkpoint blockade, but given the fact that ibrutinib can have single-agent activity in relapsed and refractory cHL, a randomized study in patients who progressed on prior CPI comparing ibrutinib monotherapy with ibrutinib with CPI would be needed to firmly establish this possibility.

The most abundant cells in the surrounding inflammatory cHL infiltrate consist of CD4+ T-cells with a T helper 2 (Th2) and T regulatory (Treg) phenotype, which provide continuous CD40L and cytokine stimulation for RS cell survival and proliferation [32]. This interaction between Th2, Treg, and RS cells also facilitate immunologic escape of the RS cells by inhibiting cytotoxic T-lymphocytes and disrupting the Th1/Th2 balance. Ibrutinib can inhibit ITK, which is necessary for Th2 signaling and proliferation while Th1 cells do not appear to be affected likely due to the compensatory resting lymphocyte kinase (RLK), which is specific to the Th1 lineage [33]. Thus, blockade of ITK may induce a shift from Th2-mediated immunity to a Th1 response thus triggering a shift to a cytotoxic T-cell and effector-cell-mediated cHL cell killing and preventing immunologic escape in this disease. This effect may be further enhanced by the known Th1 enhancement with PD1 blockade. Our data did show an increase in the Th1 cell population in responders without concomitant increase in Th2 cells after introduction treatment of both ibrutinib and nivolumab, potentially suggesting that this mechanism of ibrutinib-induced immune enhancement may not be present in nonresponders and may in part explain their poor responses. Larger patient numbers would be needed to establish this possibility and its utility as a biomarker of ongoing response. It is important to note that an enhanced Th1 response to PD1 inhibitors has been associated with life-threatening immune-related adverse events at least in case reports. However, we did not encounter these potentially fatal immune-related side effects in this trial [31].

Deep immune profiling of the circulating T cell repertoire in responders and nonresponders (not exposed to nivolumab in the 200 days preceding trial enrollment) showed expression of PD-1 on functionally diverse circulating T cell subsets, including cytotoxic and helper T cells, and naïve and memory, activated, and degranulating T cells, as well as those expressing markers of exhaustion and anergy, and checkpoint molecules. Comparison between responders and nonresponders did not show significant differences in circulating T cell populations. It may be possible that differences in the tumor microenvironment that are not reflected in the circulating T cell repertoire may account for differential responses to therapy, or a higher number of subjects may be needed to discern significant differences.

There are several limitations to this study. As it is a single-arm study, it is not possible to evaluate whether combination treatment with nivolumab and ibrutinib is superior to nivolumab alone. Furthermore, the patient population with respect to their prior treatments, most notably the use of prior nivolumab, was heterogenous, thus limiting our ability to make individual conclusions on a specific subset of patients given the smaller numbers in these subsets (e.g., nivolumab-naïve vs. nivolumab-experienced). Finally, as patients were allowed to proceed to either autologous or allogeneic transplant on this trial, censoring at the time of transplant further limited the determination of durability of responses of the ibrutinib and nivolumab combination.

## 5. Conclusions

Combination treatment with nivolumab and ibrutinib therapy was generally tolerated and achieved responses in heavily pretreated patients with cHL including in some patients who had received prior CPI therapy. Larger studies of the use of this combination in cHL in both CPI-naïve patients and patients previously treated with CPI are warranted.

## Figures and Tables

**Table 1:** Patient Characteristics.

Demographics	*N* = 17
Age, years, median (range)	40 (20–84)
Sex, no (%)	
Male	8 (47.1%)
Female	9 (52.9%)
Race, no (%)	
White	16 (94.1%)
Black or African American	1 (5.9%)
Diagnosis	
Classical Hodgkin’s lymphoma	17 (100%)
Prior lines of treatment, median (range)	5 (1–8)
Prior combination chemotherapy *N* (%) ABVD AVD + BV AVD other	15 (88.2%) 12 (70.6%) 1 (5.9%) 1 (5.9%) 1 (5.9%)
Prior autologous stem cell transplant, *N* (%)	8 (47.1%)
Prior brentuximab, *N* (%)	13 (76.5%)
Prior nivolumab, *N* (%)	10 (58.8%)

Abbreviations: ABVD = Adriamycin, bleomycin, vinblastine, dacarbazine; AVD = Adriamycin, vinblastine, Dacarbazine; BV = brentuximab.

**Table 2:** Response and Outcomes.

Best Response	*N* = 17
Overall response (95% CI)	52.9 (31.0–73.8)
Complete response (95% CI)	29.4 (12.9–53.4)
Partial response (95% CI)	23.5 (9.0–47.7)
Stable disease (95% CI)	23.5 (9.0–47.5)
Progressive disease (95% CI)	5.9 (0–28.9)
Not evaluable due to toxicity (95% CI)	11.7 (2.0–35.6)
Off-treatment Reason	*N* = 16
Disease progression (percent)	7 (43.8%)
Adverse event (percent)	4 (25.0%)
Auto-HCT transplant (percent)	2 (12.5%)
Allo-HCT transplant (percent)	2 (12.5%)
Patient withdrawal	1 (6.3%)
Progression-free survival	
Number of events	7
Number censored	10
Median	17.3
Median follow-up (months)	8.9
Duration of response	
Number of events	3
Number censored	7
Median	20.2
Median follow-up (months)	6.1

Abbreviations: HCT, hematopoietic stem cell transplant; CI, confidence interval.

**Table 3:** Treatment-related Adverse Events in ≥ 10% of pts.

	Any Grade	Grade ≥ 3
Anemia	6 (35)	0 (0)
Lymphopenia	6 (35)	2 (12)
Fatigue	5 (29)	0 (0)
Thrombocytopenia	5 (29)	0 (0)
Myalgia	5 (29)	0 (0)
Rash	4 (24)	3 (18)
Hypertension	4 (24)	0 (0)
Blood and lymphatic system disorders	3 (18)	0 (0)
Gastroesophageal reflux	3 (18)	0 (0)
Fever	3 (18)	0 (0)
Dysgeusia	3 (18)	0 (0)
Diarrhea	2 (12)	0 (0)
Dyspepsia	2 (12)	0 (0)
Dysphagia	2 (12)	0 (0)
Nausea	2 (12)	0 (0)
Emesis	2 (12)	0 (0)
Urinary tract infection	2 (12)	1 (6)
Ecchymosis	2 (12)	0 (0)
AST increased	2 (12)	0 (0)
Neutropenia	2 (12)	0 (0)
Weight gain	2 (12)	0 (0)
Leukopenia	2 (12)	0 (0)
Hematuria	2 (12)	1 (6)
Pruritis	2 (12)	0 (0)
Skin and subcutaneous disorders	2 (12)	1 (6)

## Data Availability

For all original data, please contact L.A. (lapo.alinari@osumc.edu).

## Supplementary Materials

The following supporting information can be downloaded at: https://www.mdpi.com/article/10.3390/cancers15051437/s1, Table S1. Comparison of the median percentage of PD-1+ cells in T cell subsets of the subjects that were not exposed to nivolumab for at least 200 days before enrollment on the trial, Cycle 1, Day 1. Table S2. Comparison of the median percentages of cells expressing the designated immune markers among CD8+/PD-1+ T cells in the subjects that were not exposed to nivolumab for at least 200 days before enrollment on the trial, Cycle 1, Day 1. Table S3. Comparison of the median percentages of cells expressing the designated immune markers among CD4+/PD-1+ T cells in the subjects that were not exposed to nivolumab for at least 200 days before enrollment on the trial, Cycle 1, Day 1. Table S4. Comparison of the median percentages of T cells expressing the designated immune markers between all responders and all non-responders, Cycle 1, Day 1. Table S5. Comparison of the median percentages of T cells expressing the designated immune markers between all responders and all non-responders, Cycle 4, Day 1. Table S6. Comparison of the median percentages of T cells expressing the designated immune markers between all responders and all non-responders, Cycle 4, Day 1. Table S7. Antibodies used for mass cytometry. Figure S1. Quantification of the average PD1 positive cells between responders (*n* = 7) and non-responders (*n* = 3) at baseline in all T-cells (left), CD8+ T-cells (middle) and CD4+ T-cells (right). Each dot represents an individual patient. Figure S2. Representative percentage of CD4+/PD-1+ cells on C1D1 (left) and analysis of CTLA-4 expression in CD4+/PD-1+ T cells (right) showed a trend towards higher percentage of CTLA4+ in non-responders. *p*-value 0.068 (Wilcoxon) and 0.09 (*t*-test). Center lines show the medians; box limits indicate the 25th and 75th percentiles; whiskers extend 1.5 times the interquartile range from the 25th and 75th percentiles, outliers are represented by dots. *n* = 7 (responders) and 3 (non-responders). Figure S3. Quantification of the average Th1 (left, CD4+Tbet+) and Th2 (CD4+GATA3+) cells at the indicated time points in responders (blue, *n* = 9) and non-responders (red, *n* = 4). Each dot represents an individual patient.

## References

[1] Epperla N., Hamadani M. (2021). Double-refractory Hodgkin lymphoma: Tackling relapse after brentuximab vedotin and checkpoint inhibitors. Hematology.

[2] Moskowitz A.J., Herrera A.F., Beaven A.W. (2019). Relapsed and Refractory Classical Hodgkin Lymphoma: Keeping Pace With Novel Agents and New Options for Salvage Therapy. Am. Soc. Clin. Oncol. Educ. Book.

[3] Broccoli A., Zinzani P.L. (2019). The role of transplantation in Hodgkin lymphoma. Br. J. Haematol..

[4] Chen R., Gopal A.K., Smith S.E., Ansell S.M., Rosenblatt J.D., Savage K.J., Connors J.M., Engert A., Larsen E.K., Huebner D. (2016). Five-year survival and durability results of brentuximab vedotin in patients with relapsed or refractory Hodgkin lymphoma. Blood.

[5] Chen R., Zinzani P.L., Fanale M.A., Armand P., Johnson N.A., Brice P., Radford J., Ribrag V., Molin D., Vassilakopoulos T.P. (2017). Phase II Study of the Efficacy and Safety of Pembrolizumab for Relapsed/Refractory Classic Hodgkin Lymphoma. J. Clin. Oncol..

[6] Chen R., Zinzani P.L., Lee H.J., Armand P., Johnson N.A., Brice P., Radford J., Ribrag V., Molin D., Vassilakopoulos T.P. (2019). Pembrolizumab in relapsed or refractory Hodgkin lymphoma: 2-year follow-up of KEYNOTE-087. Blood.

[7] Armand P., Engert A., Younes A., Fanale M., Santoro A., Zinzani P.L., Timmerman J.M., Collins G.P., Ramchandren R., Cohen J.B. (2018). Nivolumab for Relapsed/Refractory Classic Hodgkin Lymphoma after Failure of Autologous Hematopoietic Cell Transplantation: Extended Follow-Up of the Multicohort Single-Arm Phase II CheckMate 205 Trial. J. Clin. Oncol..

[8] Kuruvilla J., Ramchandren R., Santoro A., Paszkiewicz-Kozik E., Gasiorowski R., Johnson N.A., Fogliatto L.M., Goncalves I., de Oliveira J.S.R., Buccheri V. (2021). Pembrolizumab versus brentuximab vedotin in relapsed or refractory classical Hodgkin lymphoma (KEYNOTE-204): An interim analysis of a multicentre, randomised, open-label, phase 3 study. Lancet Oncol..

[9] Alinari L., Quinion C., Blum K.A. (2015). Bruton’s tyrosine kinase inhibitors in B-cell non-Hodgkin’s lymphomas. Clin. Pharmacol. Ther..

[10] Zhu S., Gokhale S., Jung J., Spirollari E., Tsai J., Arceo J., Wu B.W., Victor E., Xie P. (2021). Multifaceted Immunomodulatory Effects of the BTK Inhibitors Ibrutinib and Acalabrutinib on Different Immune Cell Subsets—Beyond B Lymphocytes. Front. Cell Dev. Biol..

[11] Long M., Beckwith K., Do P., Mundy B.L., Gordon A., Lehman A.M., Maddocks K.J., Cheney C., Jones J.A., Flynn J.M. (2017). Ibrutinib treatment improves T cell number and function in CLL patients. J. Clin. Investig..

[12] Parry H.M., Mirajkar N., Cutmore N., Zuo J., Long H., Kwok M., Oldrieve C., Hudson C., Stankovic T., Paneesha S. (2019). Long-Term Ibrutinib Therapy Reverses CD8(+) T Cell Exhaustion in B Cell Chronic Lymphocytic Leukaemia. Front. Immunol..

[13] Davis J.E., Handunnetti S.M., Ludford-Menting M., Sharpe C., Blombery P., Anderson M.A., Roberts A.W., Seymour J.F., Tam C.S., Ritchie D.S. (2020). Immune recovery in patients with mantle cell lymphoma receiving long-term ibrutinib and venetoclax combination therapy. Blood Adv..

[14] Dubovsky J.A., Beckwith K.A., Natarajan G., Woyach J.A., Jaglowski S., Zhong Y., Hessler J.D., Liu T.M., Chang B.Y., Larkin K.M. (2013). Ibrutinib is an irreversible molecular inhibitor of ITK driving a Th1-selective pressure in T lymphocytes. Blood.

[15] Hassenruck F., Knodgen E., Gockeritz E., Midda S.H., Vondey V., Neumann L., Herter S., Klein C., Hallek M., Krause G. (2018). Sensitive Detection of the Natural Killer Cell-Mediated Cytotoxicity of Anti-CD20 Antibodies and Its Impairment by B-Cell Receptor Pathway Inhibitors. BioMed Res. Int..

[16] Da Roit F., Engelberts P.J., Taylor R.P., Breij E.C., Gritti G., Rambaldi A., Introna M., Parren P.W., Beurskens F.J., Golay J. (2015). Ibrutinib interferes with the cell-mediated anti-tumor activities of therapeutic CD20 antibodies: Implications for combination therapy. Haematologica.

[17] Feng M., Chen J.Y., Weissman-Tsukamoto R., Volkmer J.P., Ho P.Y., McKenna K.M., Cheshier S., Zhang M., Guo N., Gip P. (2015). Macrophages eat cancer cells using their own calreticulin as a guide: Roles of TLR and Btk. Proc. Natl. Acad. Sci. USA.

[18] Fernandez-Vega I., Quiros L.M., Santos-Juanes J., Pane-Foix M., Marafioti T. (2015). Bruton’s tyrosine kinase (Btk) is a useful marker for Hodgkin and B cell non-Hodgkin lymphoma. Virchows Arch..

[19] Martin P., Salas C., Provencio M., Abraira V., Bellas C. (2011). Heterogeneous expression of Src tyrosine kinases Lyn, Fyn and Syk in classical Hodgkin lymphoma: Prognostic implications. Leuk. Lymphoma.

[20] Hamadani M., Balasubramanian S., Hari P.N. (2015). Ibrutinib in Refractory Classic Hodgkin’s Lymphoma. N. Engl. J. Med..

[21] Badar T., Astle J., Kakar I.K., Zellner K., Hari P.N., Hamadani M. (2020). Clinical activity of ibrutinib in classical Hodgkin lymphoma relapsing after allogeneic stem cell transplantation is independent of tumor BTK expression. Br. J. Haematol..

[22] Cheson B.D., Fisher R.I., Barrington S.F., Cavalli F., Schwartz L.H., Zucca E., Lister T.A., Alliance A.L., Lymphoma G., Eastern Cooperative Oncology G. (2014). Recommendations for initial evaluation, staging, and response assessment of Hodgkin and non-Hodgkin lymphoma: The Lugano classification. J. Clin. Oncol..

[23] Osa A., Uenami T., Koyama S., Fujimoto K., Okuzaki D., Takimoto T., Hirata H., Yano Y., Yokota S., Kinehara Y. (2018). Clinical implications of monitoring nivolumab immunokinetics in non-small cell lung cancer patients. JCI Insight.

[24] Advani R.H., Moskowitz A.J., Bartlett N.L., Vose J.M., Ramchandren R., Feldman T.A., LaCasce A.S., Christian B.A., Ansell S.M., Moskowitz C.H. (2021). Brentuximab vedotin in combination with nivolumab in relapsed or refractory Hodgkin lymphoma: 3-year study results. Blood.

[25] Herrera A.F., Chen R.W., Palmer J., Tsai N.-C., Mei M., Popplewell L.L., Nademanee A.P., Nikolaenko L., McBride K., Ortega R. (2019). PET-Adapted Nivolumab or Nivolumab Plus ICE As First Salvage Therapy in Relapsed or Refractory Hodgkin Lymphoma. Blood.

[26] Armand P., Lesokhin A., Borrello I., Timmerman J., Gutierrez M., Zhu L., Popa McKiver M., Ansell S.M. (2021). A phase 1b study of dual PD-1 and CTLA-4 or KIR blockade in patients with relapsed/refractory lymphoid malignancies. Leukemia.

[27] Diefenbach C.S., Hong F., Ambinder R.F., Cohen J.B., Robertson M.J., David K.A., Advani R.H., Fenske T.S., Barta S.K., Palmisiano N.D. (2020). Ipilimumab, nivolumab, and brentuximab vedotin combination therapies in patients with relapsed or refractory Hodgkin lymphoma: Phase 1 results of an open-label, multicentre, phase 1/2 trial. Lancet Haematol..

[28] Younes A., Brody J., Carpio C., Lopez-Guillermo A., Ben-Yehuda D., Ferhanoglu B., Nagler A., Ozcan M., Avivi I., Bosch F. (2019). Safety and activity of ibrutinib in combination with nivolumab in patients with relapsed non-Hodgkin lymphoma or chronic lymphocytic leukaemia: A phase 1/2a study. Lancet Haematol..

[29] Jain N., Senapati J., Thakral B., Ferrajoli A., Thompson P.A., Burger J.A., Basu S., Kadia T.M., Daver N.G., Borthakur G. (2022). A Phase 2 Study of Nivolumab Combined with Ibrutinib in Patients with Diffuse Large B-cell Richter Transformation of CLL. Blood Adv..

[30] Manson G., Brice P., Herbaux C., Bouabdallah K., Antier C., Poizeau F., Dercle L., Houot R. (2020). Efficacy of anti-PD1 re-treatment in patients with Hodgkin lymphoma who relapsed after anti-PD1 discontinuation. Haematologica.

[31] Fedorova L.V., Lepik K.V., Mikhailova N.B., Kondakova E.V., Zalyalov Y.R., Baykov V.V., Babenko E.V., Kozlov A.V., Moiseev I.S., Afanasyev B.V. (2021). Nivolumab discontinuation and retreatment in patients with relapsed or refractory Hodgkin lymphoma. Ann. Hematol..

[32] Schreck S., Friebel D., Buettner M., Distel L., Grabenbauer G., Young L.S., Niedobitek G. (2009). Prognostic impact of tumour-infiltrating Th2 and regulatory T cells in classical Hodgkin lymphoma. Hematol. Oncol..

[33] Berglof A., Hamasy A., Meinke S., Palma M., Krstic A., Mansson R., Kimby E., Osterborg A., Smith C.I. (2015). Targets for Ibrutinib Beyond B Cell Malignancies. Scand. J. Immunol..

