# Supplementary material for: A Phase 2 Trial of Ibrutinib and Nivolumab in Patients with Relapsed or Refractory Classical Hodgkin’s Lymphoma
Source: Cancers (Basel). 2023 Feb 24;15(5):1437. doi: 10.3390/cancers15051437 (PMC10000669; doi:10.3390/cancers15051437)
Supplement: Supplementary file 1 [file cancers-15-01437-s001.zip › cancers-2158844-supplementary.pdf]

Table S1. Comparison of the median percentage of PD-1+ cells in T cell subsets of the subjects that were not exposed to nivolumab for at least 200 days before enrollment on the trial, Cycle 1, Day 1.

Immune markers	Responder median (n=7)	Nonresponder median (n=3)	Wilcoxon p-value	T-test p-value
% PD1 in CD3	23.24	19.38	0.49412	0.63
% PD-1 in CD4	22.26	18.34	1	0.68
% PD-1 in CD8	30.354	22.913	0.49412	0.55
% PD-1 in CD4+/CD8+	39.4	32.09	0.81971	0.84
%PD-1 in Perf+/GrB+/CD4+	38.41	37.5	1	0.69
% PD-1 in Perf+/GrB-/CD4+	46.711	27.27	0.17146	0.26
% PD-1 in Perf+/GrB-/CD8+	39.479	30	0.36193	0.29
% PD-1 in Perf+/Grb+/CD8+	40.706	32.56	0.36193	0.38
% PD-1 in CD4+/HLA-DR+	53.34	26.67	0.36193	0.18
% PD-1 in CD8+/HLADR+	35.69	36.39	1	0.7
% PD-1 in CD28+/CD4+	23.024	18.85	0.81971	0.65
% PD-1 in CD28+/CD8+	19.712	17.72	0.49412	0.87
% PD-1 in CD25+/CD4+	27.521	17.56	0.49412	0.57
% PD-1 in CD25+/CD8+	19.601	15.84	0.6485	0.57
% PD-1 in CD4+/FoxP3+	32.325	29.962	0.81971	0.68
% PD-1 in CD8+/FoxP3+	45.455	25	0.49412	0.46
% PD-1 in CD4+/Tbet+	39.631	28.57	0.49281	0.7
% PD-1 in CD8+/Tbet+	36.684	33.93	0.49412	0.49
% PD-1 in CD4+/GATA3+	38.89	43.93	0.65	0.55
% PD-1 in CD8+/GATA3+	28.767	40.26	0.36193	0.27
% PD-1 in CD4+/Fas+	35.881	25.81	0.81971	0.66
% PD-1 in CD8+/Fas+	34.003	36.45	1	0.76
% PD-1 in NKG2D+/CD4+	45.61	53.623	0.49412	0.28
% PD-1 in NKG2D+/CD8+	38.673	29.6	0.49412	0.38
% PD-1 in CD45RA+/CD4+	10.227	13.71	0.73166	0.44
% PD-1 in CD45RA+/CD8+	19.53	10.98	0.49412	0.53
% PD-1 in CD45RO+/CD4+	35.728	20.18	0.6485	0.43
% PD-1 in CD45RO+/CD8+	34.791	35.97	1	0.54
% PD-1 in CD62L+/CD4+	19.718	19.46	0.81971	0.9
% PD-1 in CD62L+/CD8+	21.839	20.6	0.6485	0.69
% PD-1 in PD-L1+/CD4+	31.974	19.05	0.81971	0.7
% PD-1 in PD-L1+/CD8+	37.412	24.24	0.49412	0.31
% PD-1 in LAG3+/CD4+	40.738	25.89	1	0.89
% PD-1 in LAG3+/CD8+	43.966	33.33	0.49412	0.75
% PD-1 in CTLA4+/CD4+	40.367	50	0.81971	0.83
% PD-1 in CTLA4+/CD8+	43.651	12.7	0.36193	0.38
% PD-1 in CD57+/CD4+	45.455	74.157	0.49412	0.25
% PD-1 in CD57+/CD8+	44.702	24.26	0.36193	0.4
% PD-1 in Tim3+/CD4+	44.186	52.63	0.25445	0.38

% PD-1 in Tim3+/CD8+	42.739	15.79	0.17146	0.17
% PD-1 in Perf Hi/Granzyme B+/CD4+	53.571	33.333	1	0.91
% PD-1 in Perf Hi/Granzyme B+/CD8+	41.387	39.604	0.49412	0.45

Table S2. Comparison of the median percentages of cells expressing the designated immune markers among CD8+/PD-1+ T cells in the subjects that were not exposed to nivolumab for at least 200 days before enrollment on the trial, Cycle 1, Day 1.

Immune marker	Responder median (n=7)	Nonresponder median (n=3)	Wilcoxon p-value	T-test p-value
FoxP3+	12.1	7.69	0.25445	0.3
Tbet+	15.962	7.22	0.36193	0.32
GATA3+	1.48	1.03	1	0.94
Perf+/GrB+	17.91	29.87	0.56764	0.58
Perf+ Hi/GrB+	1.097	0.323	0.81752	0.81
Perf+/GrB-	5.105	3.09	0.6485	0.47
HLA-DR+	28.36	32.99	1	0.94
CD28+	94.72	92.78	0.6485	0.72
CD25+	43.279	51.13	0.17146	0.14
CD95+/Fas+	96.244	78.96	1	0.79
NKG2D+	3.767	1.6	0.25445	0.69
CD45RA+	7.069	2.606	0.49412	0.58
CD45RO+	96.358	97.988	0.6485	0.9
CD62L+	51.6	38.363	0.49412	0.72
PD-L1+	5.778	2.378	0.49412	0.54
PD-L2+	6.52	10.105	0.17146	0.29
LAG3+	0.288	2.26	0.6485	0.97
CTLA4+	0.274	1.13	0.06825	0.09
CD57+	3.727	2.972	1	0.55
Tim-3+	2.122	2.04	1	0.61

Table S3. Comparison of the median percentages of cells expressing the designated immune markers among CD4+/PD-1+ T cells in the subjects that were not exposed to nivolumab for at least 200 days before enrollment on the trial, Cycle 1, Day 1.

Immune marker	Responder median (n=7)	Nonresponder median (n=3)	Wilcoxon p-value	T-test p-value
FoxP3+	12.1	7.69	0.25445	0.3
Tbet+	15.962	7.22	0.36193	0.32
GATA3+	1.48	1.03	1	0.94
Perf+ GrB+	17.91	29.87	0.56764	0.58
Perf+ HI GrB+	1.097	0.323	0.81752	0.81
Perf+ GrB-	5.105	3.09	0.6485	0.47
HLA-DR+	28.36	32.99	1	0.94
CD28+	94.72	92.78	0.6485	0.72
CD25+	43.279	51.13	0.17146	0.14
CD95 Fas	96.244	78.96	1	0.79
NKG2D	3.767	1.6	0.25445	0.69
CD45RA	7.069	2.606	0.49412	0.58
CD45RO	96.358	97.988	0.6485	0.9
CD62L	51.6	38.363	0.49412	0.72
PD-L1	5.778	2.378	0.49412	0.54
PD-L2	6.52	10.105	0.17146	0.29
LAG3	0.288	2.26	0.6485	0.97
CTLA4	0.274	1.13	0.06825	0.09
CD57	3.727	2.972	1	0.55
Tim-3	2.122	2.04	1	0.61

Table S4. Comparison of the median percentages of T cells expressing the designated immune markers between all responders and all non-responders, Cycle 1, Day 1.

Immune marker	Responder median (n=9)	Nonresponder median (n=5)	Wilcoxon p-value	T-test p-value
FoxP3+/CD4+	3.124	1.776	0.0455	0.17
Tbet+/CD4+	3.33	2.073	0.50499	0.44
GATA3+/CD4+	0.494	0.074	0.1096	0.27
Perf+/GrB+/CD4+	4.741	1.049	0.50499	0.27
Perf+ HI/GrB+/CD4+	0.188	0.0323	0.28077	0.15
Perf+/GrB-/CD4+	1.111	0.814	0.42371	0.41
HLA-DR+/CD4+	4.572	7.934	0.14247	0.15
CD28+/CD4+	21.746	37.327	0.28612	0.47
CD25+/CD4+	12.778	26.971	0.35065	0.31
CD95+/CD4+	15.958	20.057	0.23014	0.31
NKG2D+/CD4+	1.55	0.974	0.5938	0.52
CD45RA+/CD4+	5.774	0.817	0.14247	0.15
CD45RO+/CD4+	17.619	41.877	0.23014	0.3
CD62L+/CD4+	7.111	8.542	1	0.73
PD-L1+/CD4+	1.885	1.42	0.78973	0.34
PD-L2+/CD4+	1.03	2.414	0.35065	0.17
LAG3+/CD4+	0.125	0.212	0.68883	0.68
CTLA4+/CD4+	0.233	0.378	0.23014	0.18
CD57+/CD4+	0.749	0.516	0.42371	0.38
Tim-3+/CD4+	2.308	0.72	0.02341	0.055
FoxP3+/CD8+	3.492	4.957	0.50499	0.26
Tbet+/CD8+	11.291	3.466	0.28612	0.25
GATA3+/CD8+	1.579	0.465	0.5938	0.53
Perf+/GrB+/CD8+	20.961	18.595	0.78973	0.8
Perf+ HI/GrB+/CD8+	6.977	6.603	0.78973	0.69
Perf+/GrB-/CD8+	14.808	5.209	0.35065	0.38
HLA-DR+/CD8+	8.693	7.7	0.89393	0.99
CD28+/CD8+	16.032	11.975	0.78973	0.44
CD25+/CD8+	9.302	6.725	0.78973	0.44
CD95+/CD8+	29.133	17.765	0.50499	0.33
NKG2D+/CD8+	9.302	5.329	0.5938	0.94
CD45RA+/CD8+	16.849	12.876	0.18242	0.2
CD45RO+/CD8+	18.413	24.056	0.06195	0.07
CD62L+/CD8+	18.605	15.909	0.18242	0.14
PD-L1+/CD8+	1.684	0.446	0.06195	0.15
PD-L2+/CD8+	1.056	1.102	0.89393	0.61
LAG3+/CD8+	0.448	0.243	1	0.56
CTLA4+/CD8+	0.68	1.149	0.50499	0.51

CD57+/CD8+	6.732	6.816	0.68916	0.74
Tim-3+/CD8+	2.326	1.406	0.08304	0.19

Table S5. Comparison of the median percentages of T cells expressing the designated immune markers between all responders and all non-responders, Cycle 4, Day 1.

Immune marker	Responder median (n=8)	Nonresponder median (n=4)	Wilcoxon p-value	T-test p-value
FoxP3+/CD4+	3.384	3.11	0.93232	0.65
Tbet+/CD4+	3.4745	1.755	0.4447	0.2
GATA3+/CD4+	0.296	0.225	0.73365	0.4
Perf+/GrB+/CD4+	5.8775	3.2455	0.26961	0.21
Perf+ HI/GrB+ CD4+	2.6165	0.0709	0.0508	0.12
Perf+/GrB-/CD4+	0.64	0.729	0.67113	0.17
HLA-DR+/CD4+	4.772	9.445	0.10664	0.051
CD28+/CD4+	14.0645	40.119	0.35024	0.4
CD25+/CD4+	8.2555	22.2365	0.26961	0.48
CD95+/CD4+	12.95	24.461	0.35024	0.37
NKG2D+/CD4+	2.054	0.872	0.26961	0.18
CD45RA+/CD4+	7.8805	4.764	0.20273	0.21
CD45RO+/CD4+	11.7965	41.9895	0.26961	0.2
CD62L+/CD4+	8.6185	12.1615	0.67113	0.77
PD-L1+/CD4+	1.5115	1.2265	0.93232	0.87
PD-L2+/CD4+	1.7025	3.0295	0.10664	0.07
LAG3+/CD4+	0.126	0.2491	0.79752	0.56
CTLA4+/CD4+	0.27	0.367	0.67113	0.55
CD57+/CD4+	0.963	0.7	0.26961	0.34
Tim-3+/CD4+	1.329	0.7605	0.26961	0.2
FoxP3+/CD8+	4.075	4.6645	0.79891	0.22
Tbet+/CD8+	28.649	3.068	0.14884	0.08
GATA3+/CD8+	0.9285	0.6905	0.4447	0.36
Perf+/GrB+/CD8+	29.4905	23.538	0.67113	0.7
Perf+ HI/GrB+/CD8+	21.912	10.2415	0.20273	0.31
Perf+/GrB-/CD8+	10.306	9.6285	0.93232	0.6
HLA-DR+/CD8+	9.082	11.4115	0.55221	0.41
CD28+/CD8+	17.696	15.6425	0.79891	0.55
CD25+/CD8+	6.5735	10.9625	0.4447	0.23
CD95+/CD8+	26.8285	26.9235	0.79891	0.67
NKG2D+/CD8+	15.669	9.384	0.93232	0.83
CD45RA+/CD8+	20.1525	11.568	0.26961	0.19
CD45RO+/CD8+	17.424	26.5885	0.02186	0.03
CD62L+/CD8+	25.059	18.1145	0.4447	0.52
PD-L1+/CD8+	1.479	0.835	0.93232	0.33
PD-L2+/CD8+	1.2	1.4485	0.55221	0.71
LAG3+/CD8+	0.293	0.637	0.10664	0.06
CTLA4+/CD8+	0.5805	1.3945	0.4447	0.14
CD57+/CD8+	8.5715	9.394	1	0.9

Tim-3+/CD8+	4.285	3.1505	0.55221	0.41

Table S6. Comparison of the median percentages of T cells expressing the designated immune markers between all responders and all non-responders, Cycle 4, Day 1.

Immune marker	Responder median (n=3)	Nonresponder median (n=1)
FoxP3+/CD4+	2.182	3.256
Tbet+/CD4+	0.919	1.598
GATA3+/CD4+	0.364	0.109
Perf+/GrB+/CD4+	0.727	0.287
Perf+ HI/GrB+/CD4+	0.0249	0.029
Perf+/GrB-/CD4+	0.647	1.125
HLA-DR+/CD4+	3.05	4.506
CD28+/CD4+	13.893	31.723
CD25+/CD4+	8.389	19.805
CD95+/CD4+	13.064	29.543
NKG2D+/CD4+	2.545	0.35
CD45RA+/CD4+	10.146	15.214
CD45RO+/CD4+	12.997	30.488
CD62L+/CD4+	8.123	18.586
PD-L1+/CD4+	0.368	0.339
PD-L2+ CD4+	1.257	0.876
LAG3+/CD4+	0	0.178
CTLA4+/CD4+	0.0332	0.441
CD57+/CD4+	0.239	0.223
Tim-3+/CD4+	0.488	0.501
FoxP3+/CD8+	1.558	3.091
Tbet+/CD8+	14.545	1.578
GATA3+/CD8+	1.091	0.194
Perf+/GrB+/CD8+	9.818	1.433
Perf+ HI/GrB+/CD8+	6.182	0.201
Perf+/GrB-/CD8+	24.35	16.455
HLA-DR+/CD8+	5.902	10.931
CD28+/CD8+	11.353	11.66
CD25+/CD8+	2.182	3.236
CD95+/CD8+	9.326	12.611
NKG2D+/CD8+	9.9	8.081
CD45RA+/CD8+	9.383	14.046
CD45RO+/CD8+	12.378	21.454
CD62L+/CD8+	16.86	15.76
PD-L1+/CD8+	0.147	0.152
PD-L2+/CD8+	0.177	0.457
LAG3+/CD8+	0.192	0.131
CTLA4+/CD8+	0.365	0.421

CD57+/CD8+	5.338	3.485
Tim-3+/CD8+	4	1.219

Table S7. Antibodies used for mass cytometry.

Metal	Mass	Target	Clone	Vendor
Y	89	CD235	HIR2	Biolegend
In	113	CD3	UCHT1	Biolegend
In	115	CD45	HI30	Biolegend
La	139	Granzyme B	QA16A02	Biolegend
Ce	140	cPARP	F21-852	BD Biosciences
Nd	142	CD7	CD7-6B7	Biolegend
Nd	143	T-Bet	eBio4B10	eBioscience
Nd	144	CD95 (Fas)	DX2	Biolegend
Nd	145	CD4	RPA-T4	Biolegend
Nd	146	CD8a	RPA-T8	Biolegend
Sm	147	CD56	HCD56	Biolegend
Nd	148	CD62L	DREG-56	Biolegend
Sm	149	CD127	A019D5	Fluidigm
Nd	150	CD45RO	UCHL1	Biolegend
Eu	151	CD14	M5E2	Fluidigm
Sm	152	CD33	WM53	Biolegend
Eu	153	HLA-DR	L243	Biolegend
Sm	154	TIM-3	F382E2	Biolegend
Gd	155	CD279 (PD-1)	EH12.2H7	Biolegend
Gd	157	CD161	HP-3G10	Biolegend
Gd	158	CD45RA	HI100	Biolegend
Tb	159	GATA3	16E10A23	Biolegend
Gd	160	CD28	CD28.2	Fluidigm
Dy	161	CD16	3G8	Biolegend
Dy	162	Foxp3	PCH101	Fluidigm
Dy	163	Perforin	B-D48	Abcam
Dy	164	CD15	W6D3	Biolegend
Er	166	NKG2D	1D11	Biolegend
Tm	169	CD25	2A3	Fluidigm
Er	170	CTLA-4	14D3	Fluidigm
Yb	171	CD226	DX11	Fluidigm
Yb	172	CD273 (PD-L2)	24F.10C12	Fluidigm
Yb	173	CD19	HIB19	Biolegend
Yb	174	CD57	HNK-1	Biolegend
Lu	175	CD274 (PD-L1)	29E.2A3	Biolegend
Yb	176	LAG3	1009614	R&D Systems
Bi	209	CD11b	ICRF44	Biolegend

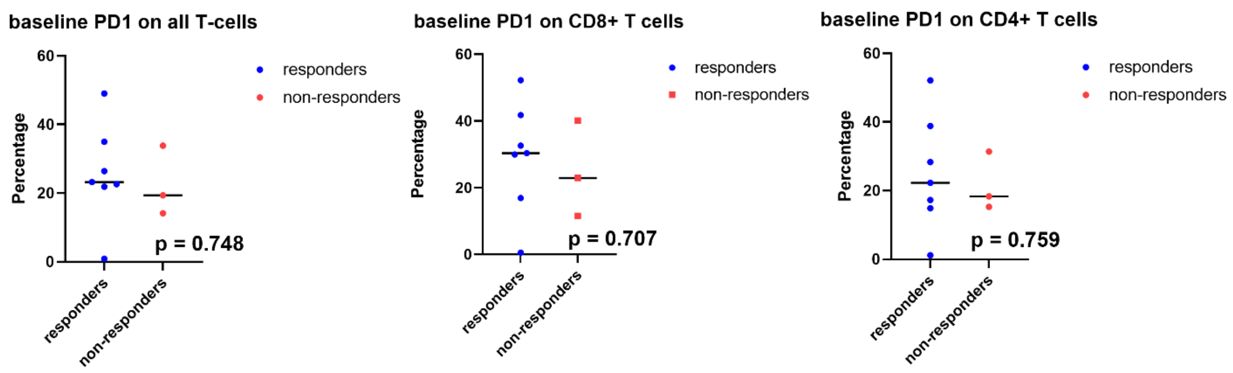

Figure S1. Quantification of the average PD1 positive cells between responders (n=7) and non-responders (n=3) at baseline in all T-cells (left), CD8+ T-cells (middle) and CD4+ T-cells (right). Each dot represents an individual patient.

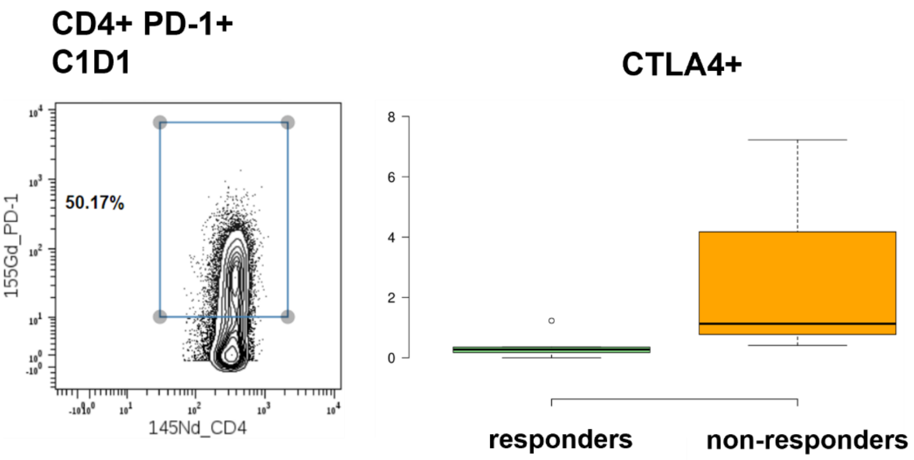

Figure S2. Representative percentage of CD4+/PD-1+ cells on C1D1 (**left**) and analysis of CTLA-4 expression in CD4+/PD-1+ T cells (**right**) showed a trend towards higher percentage of CTLA4+ in non-responders. p-value 0.068 (Wilcoxon) and 0.09 (t-test). Center lines show the medians; box limits indicate the 25th and 75th percentiles; whiskers extend 1.5 times the interquartile range from the 25th and 75th percentiles, outliers are represented by dots. n = 7 (responders) and 3 (non-responders).

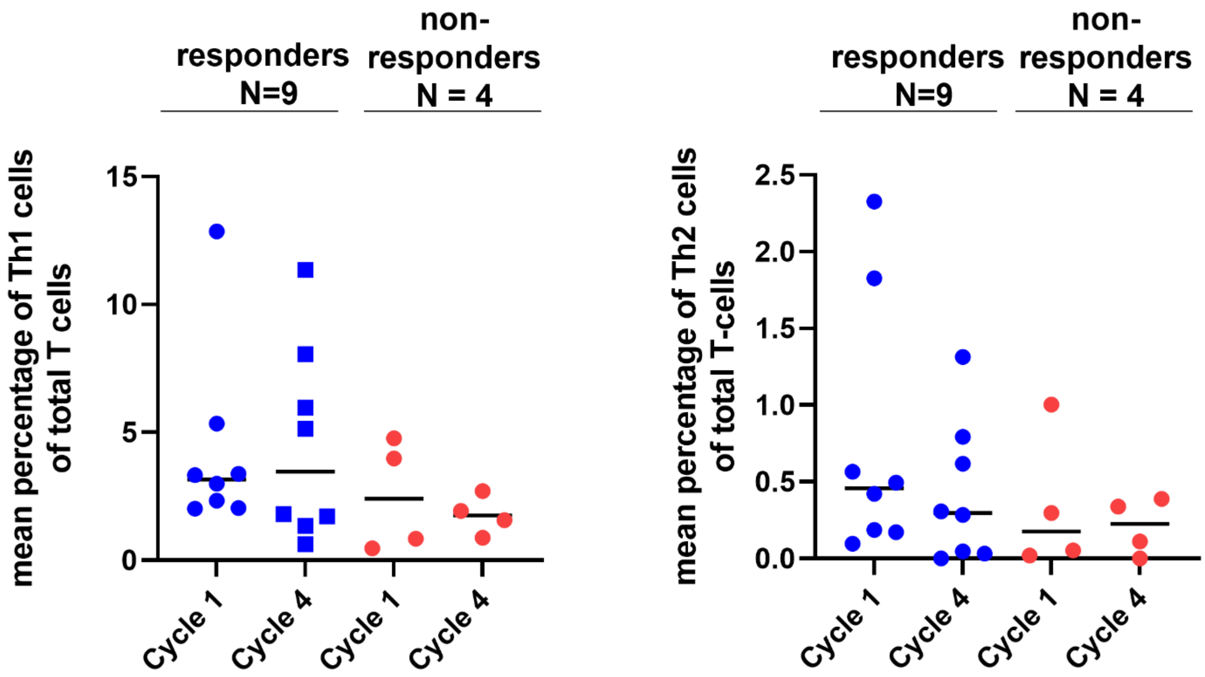

Figure S3. Quantification of the average Th1 (left, CD4+Tbet+) and Th2 (CD4+GATA3+) cells at the indicated time points in responders (blue, n=9) and non-responders (red, n=4). Each dot represents an individual patient.
